# Screening of Alkali-Resistant Cellulolytic Bacteria for Improving the Nutritional Value of Ammoniated Wheat Straw: Identification of Optimal Strain and Storage Duration

**DOI:** 10.3390/ani16142138

**Published:** 2026-07-09

**Authors:** Guofang Chen, Haichao Yan, Jiawei Lu, Luyao Zhang, Qiang Liu, Cong Wang, Gang Guo, Lei Chen, Wenjie Huo

**Affiliations:** 1 College of Animal Sciences, Shanxi Agricultural University, 1 Minxian Street, Jinzhong 030801, China; chengf0036@163.com (G.C.); 16634236243@163.com (H.Y.); ljw15735933602@163.com (J.L.); liuqiangabc@163.com (Q.L.); wangdx0321@163.com (C.W.); gggg1984@163.com (G.G.); cl1016zj@126.com (L.C.); 2 Heilongjiang Animal Husbandry General Station, 243 Haping Road, Xiangfang District, Harbin 150069, China; zly13125997162@163.com

**Keywords:** alkali-resistant cellulolytic bacteria, ammoniated wheat straw, in vitro ruminal fermentation, methane production, *Bacillus pumilus*

## Abstract

Wheat straw (WS) is an abundant crop residue with considerable potential as a ruminant feed; however, its utilization is severely constrained by a recalcitrant lignocellulosic structure. This study evaluated the effects of five alkali-resistant cellulolytic bacterial inoculants on the structural carbohydrate composition and in vitro ruminal fermentation characteristics of ammoniated wheat straw. A 6 × 3 factorial arrangement was employed, with six treatments (ammoniated WS as a control and five cellulolytic bacterial strains: X67, C72, S87, D100, and X107) and three storage durations (7, 14, and 21 days). The results showed that the bacterial treatments caused moderate losses of dry matter (DM), neutral detergent fiber (NDF), acid detergent fiber (ADF), hemicellulose, and cellulose at 7 and 14 days. The X107 treatment exhibited the lowest hemicellulose content, 2.1% lower than the ammoniated control. After 48 h of in vitro incubation, all bacterial treatments significantly increased potential gas production, 48 h methane production, in vitro DM digestibility (IVDMD), and in vitro NDF digestibility (IVNDFD). The S87 treatment achieved the highest IVDMD and IVNDFD, exceeding the ammoniated control by 14.9% and 32.5%, respectively, at 7 days (*p* < 0.05). All bacterial treatments maintained relatively high total volatile fatty acid (VFA) concentrations. Furthermore, the bacterial treatments increased the relative proportions of ruminal cellulolytic microbes at 7 and 14 days. By 21 days, no significant differences were observed in DM loss or microbial proportions among treatments. These findings indicate that the application of cellulolytic bacterial additives, with appropriate selection of bacterial strain and storage duration, exerts synergistic positive effects on the feeding value of alkali-pretreated wheat straw. The S87 treatment with a 7-day storage duration proved most effective.

## Simple Summary

Wheat straw is an abundant and low-cost feed resource for ruminants, but its high fiber content limits its digestibility. Treating straw with ammonia helps break down some of these fibers. In this study, we added five different types of alkali-resistant, fiber-degrading bacteria to ammoniated wheat straw and stored them for up to 21 days. We found that these bacterial additives further improved the nutritional value of straw by breaking down more structural carbohydrates. This led to better digestibility and increased energy availability (volatile fatty acids) during lab-simulated rumen fermentation, although it also resulted in higher methane production. The best results were achieved by adding the *Bacillus pumilus* strain S87 and storing for only 7 days. This work provides an effective, low-cost biological strategy to upgrade agricultural by-products like wheat straw into a higher-quality feed, although the trade-off between improved digestibility and increased methane production warrants further investigation for environmental sustainability.

## 1. Introduction

Crop residues, particularly wheat straw (WS), represent a substantial yet underutilized feed resource for ruminant production systems worldwide [1]. Annually, billions of tonnes of cereal straws are produced as agricultural by-products, offering a renewable and cost-effective alternative to conventional forages [2]. However, the feeding value of wheat straw is inherently limited by the complex architecture of the plant cell wall, which is primarily composed of cellulose, hemicellulose, and lignin [3,4]. The crystalline nature of cellulose and the cross-linking between lignin and hemicellulose via esterified bonds create a physical barrier that restricts access by ruminal microorganisms and their hydrolytic enzymes, thereby limiting the digestibility of these recalcitrant feeds [5,6].

Pretreatment strategies are essential to disrupt the lignocellulosic matrix and enhance the subsequent enzymatic and microbial degradation of wheat straw [7]. Among chemical pretreatment methods, alkali treatment with ammonia solution has been widely demonstrated to effectively cleave ester bonds within the cell wall, improve palatability, and increase crude protein content approximately two-fold [8,9,10]. Ammoniation modifies the physical structure of cellulose by weakening inter-chain hydrogen bonds, reducing crystallinity, and promoting hemicellulose solvation [11,12]. Nevertheless, ammonia alone has limited capacity to degrade lignin, suggesting that a combined approach integrating chemical and biological pretreatments may offer synergistic advantages.

Cellulolytic bacteria are specialized microorganisms capable of degrading cellulose and, in some cases, hemicellulose through the secretion of hydrolytic enzymes such as endoglucanases, exoglucanases, and xylanases. Their application as biological additives offers an environmentally sustainable approach to enhancing the feeding value of crop residues [13,14]. Bacteria vary considerably in their cultivation requirements, pH tolerance, and substrate specificity, prompting increasing research interest in the isolation and characterization of lignocellulose-degrading strains [15]. However, the combined effects of alkali-resistant cellulolytic bacteria and ammonia pretreatment on the structural carbohydrate composition and ruminal fermentation characteristics of wheat straw have not been systematically investigated.

Although ammoniation and cellulolytic bacterial inoculation have each been shown to improve the feeding value of straw, the synergistic effects of their combination, especially the systematic screening of alkali-resistant strains and process optimization, remain underexplored. Most published studies have used commercial enzyme preparations or conventional strains without prior screening for alkali tolerance. Those strains may be inhibited in their colonization and enzyme production on ammoniated straw, which retains an alkaline environment [16,17]. Furthermore, the diversity of strain sources (e.g., various animal feces, decaying wood) and the optimal storage duration after inoculation (i.e., the time window during which the bacteria can act without excessively consuming nutrients) are critical factors for practical application, but they have not been systematically addressed.

Therefore, the objectives of this study were to (1) screen five alkali-resistant cellulolytic bacterial strains of different origins for their efficacy in degrading structural carbohydrates in ammoniated wheat straw, (2) determine the optimal storage duration for bacterial pretreatment, and (3) evaluate the effects of bacterial inoculation on in vitro ruminal gas production, methane emission, nutrient digestibility, and microbial community composition. We hypothesized that the application of alkali-resistant cellulolytic bacterial inoculants to ammoniated wheat straw would further enhance its feeding value compared with ammoniation alone.

## 2. Materials and Methods

### 2.1. Materials

Wheat straw was harvested at Wanrong County, Yuncheng, Shanxi Province, China. Following harvest, the wheat straw was chopped to a theoretical length of 2 cm using a fodder chopper. Half of the material was designated as untreated wheat straw (Control), while the remaining half was treated with 3% (*w*/*w*) ammonia solution sprayed onto the wheat straw at a rate equivalent to 50% of fresh weight (FW). After mixing thoroughly, the ammoniated wheat straw was packed into polyethylene plastic bags, sealed, and stored at ambient temperature (approximately 20 °C) for 15 days. The polyethylene bags were opened on day 15 for subsequent bacterial inoculation and laboratory analyses.

### 2.2. Alkali-Resistant Cellulolytic Bacteria Treatments

Five alkali-resistant cellulolytic bacterial strains were used in this experiment: X67 (*Bacillus subtilis*, isolated from rhinoceros faeces), C72 (*Exiguobacterium aurantiacum*, isolated from giraffe faeces), S87 (*Bacillus pumilus*, isolated from decaying wood), D100 (*Bacillus pumilus*, isolated from elephant faeces), and X107 (*Bacillus pumilus*, isolated from rhinoceros faeces). Prior to the straw inoculation experiment, each bacterial strain was cultivated separately in carboxymethylcellulose (CMC) sodium salt liquid medium. The bacterial inoculum was prepared by adding 3% (*v*/*w*) bacterial culture to the medium, followed by incubation at 35 °C with shaking at 120 rpm for 48 h. Serial dilutions (10^−1^ to 10^−8^) were prepared, and three appropriate dilutions were plated onto solid medium for aerobic incubation at 35 °C for 48 h. Colony-forming units (CFU) were enumerated using the plate count method. For each of two independent experimental runs, fifty-four sub-samples of ammoniated wheat straw (approximately 500 g each) were randomly allocated to six treatments (ammoniated control and five bacterial treatments) and three storage durations (7, 14, and 21 days), resulting in three independent biological storage replicates per treatment × storage combination (6 treatments × 3 storage durations × 3 biological replicates = 54 sub-samples per run). Each sub-sample was prepared as a separate polyethylene bag containing approximately 500 g of inoculated ammoniated straw, stored hermetically at ambient temperature for the designated duration, and processed independently thereafter. Bacterial inoculum was applied at a rate of 10^6^ CFU/g fresh weight, thoroughly mixed into the straw prior to storage. At each sampling time, sub-samples were collected, dried at 65 °C for 48 h, ground to pass a 1 mm screen, and stored for subsequent chemical analysis and in vitro ruminal fermentation.

### 2.3. In Vitro Ruminal Methane Production

Rumen fluid was collected via the permanent ruminal cannula from three ruminally fistulated Jinnan cattle (650 ± 20 kg) at 2 h before the morning feeding. The animals were fed a total mixed ration consisting of 21.5% oat hay, 17.8% Chinese wild rye, 45.8% whole corn silage, 3% soybean meal, 2.5% DDGS, 5% wheat bran, and 4.4% vitamins and minerals. The rumen fluid was pooled in equal volumes and strained through four layers of cheesecloth into pre-warmed, CO_2_-flushed thermos flasks and immediately transported to the laboratory. The artificial buffer solution was prepared according to the method described by Longland [18]. The filtered rumen fluid was mixed with artificial buffer solution at a ratio of 1:2 (*v*/*v*). The mixture was maintained in a water bath at 39 °C and continuously flushed with CO_2_ throughout the incubation period. Milled wheat straw samples (0.5 g) were weighed into pre-weighed nylon bags (pore size 38–40 µm), sealed with nylon cord, and placed into 100 mL calibrated glass syringes. Fifty millilitres of the rumen fluid–buffer mixture was introduced into each syringe, the air was expelled, and the initial gas volume was recorded. Syringes were incubated at 39 °C for 48 h, with each treatment prepared in triplicate. Blank controls (50 mL of rumen fluid-buffer mixture without substrate) were included in three replications. Gas production was recorded at 4, 8, 12, 24, and 48 h. The cumulative gas production data were fitted to the exponential model of Ørskov and McDonald [19]: Y = b(1 – e^−ct^), where Y is the gas production at time t, b is the potential gas production (mL), c is the fractional rate of gas production, and t is the incubation time (h). The 48-h methane production was determined by gas chromatography (GC-TRACE 1300, Thermo Fisher Scientific, Waltham, MA, USA) equipped with a flame ionization detector (220 °C), injection temperature of 100 °C, and column temperature of 130 °C, according to the method of Kougias [20]. Following incubation, the nylon bags were recovered, washed with running water, and dried at 65 °C for 48 h for subsequent nutrient analyses.

### 2.4. Chemical Analyses

All samples were dried at 65 °C for 48 h for dry matter (DM) determination. Organic matter (OM) was determined by ashing at 550 °C [21]. Total nitrogen (TN) was determined using the Kjeldahl method [22], and crude protein (CP) was calculated as TN multiplied by 6.25. Neutral detergent fibre (NDF), acid detergent fibre (ADF), and acid detergent lignin (ADL) were analysed according to Van Soest [23]. Hemicellulose was calculated as the difference between NDF and ADF. Cellulose content was estimated as ADF minus ADL and ash. In vitro dry matter digestibility (IVDMD) and in vitro neutral detergent fibre digestibility (IVNDFD) were calculated using the formulae described by Tilley and Terry [24]. The incubation liquid was analysed for volatile fatty acids (VFA) via gas chromatography (GC-TRACE 1300, Thermo Fisher Scientific, Waltham, MA, USA) according to Guo [25]. Ruminal cellulase activities, including carboxymethyl-cellulase, xylanase, and beta-glycosidase, were determined using the method described by Agarwal [26].

### 2.5. Microbial Quantitative Real-Time PCR Analysis

The incubation liquid was filtered through four layers of cheesecloth and centrifuged at 15,000× *g* for 5 min. DNA was extracted from the resulting pellet using a commercial kit (Tiangen Biotech, Beijing, China) according to the manufacturer’s instructions. Quantitative real-time PCR was performed using the SYBR PrimeScript RT-PCR Kit (Tiangen Biotech, Beijing, China) on a StepOne Plus real-time PCR system (Applied Biosystems, Foster City, CA, USA). The PCR primers used for quantification of total bacteria, *Ruminococcus albus*, *Ruminococcus flavefaciens*, *Fibrobacter succinogenes*, and *Butyrivibrio fibrisolvens* are listed in Table 1. Each 20 uL reaction mixture contained 10 uL of SYBR Premix Ex Taq II (2×), 0.8 uL of each forward and reverse primer (10 uM), 0.4 uL of ROX Reference Dye (50×), 1.0 uL of DNA template, and 7.0 uL of nuclease-free water. The thermal cycling conditions consisted of an initial denaturation at 95 °C for 30 s, followed by 40 cycles of 95 °C for 15 s and 60 °C for 1 min. Relative quantification was calculated according to the 2-DeltaCt method [27]: relative abundance = 2^−(Ct_target−Ct_total bacteria)^, where Ct represents the threshold cycle.

### 2.6. Statistical Analyses

The experimental design was a completely randomized 6 × 3 factorial arrangement, with treatment (6 levels: A, X67, C72, S87, D100, X107) and storage duration (3 levels: 7, 14, and 21 days) as the two fixed factors. Data were analysed using the general linear model (GLM) procedure of SAS 9.4 (SAS Institute Inc., Cary, NC, USA) with a two-way analysis of variance (ANOVA). The statistical model was: Y_ijk_ = mu + T_i_ + D_j_ + (TD)_ij_ + e_ijk_, where Y_ijk_ is the observed response variable, mu is the overall mean, T_i_ is the fixed effect of treatment i (i = 1, 2, …, 6), D_j_ is the fixed effect of storage duration j (j = 7, 14, 21 days), (TD)_ij_ is the interaction effect between treatment and storage duration, and e_ijk_ is the residual error assumed to be normally and independently distributed with mean zero and variance sigma^2^. All data are presented as least squares means with pooled standard error of the mean (SEM). When significant F-values were detected (*p* < 0.05), treatment means were separated using Duncan’s multiple range test. Main effects and interactions were considered significant at *p* < 0.05.

## 3. Results

### 3.1. Chemical Composition of Ammoniated Wheat Straw

Compared with the untreated control, ammoniation significantly (*p* < 0.05) decreased the DM, NDF, ADF, hemicellulose, and cellulose contents of WS (Table 2). ADL content was only marginally affected (*p* = 0.053). Notably, ammoniation increased CP content by 89% compared with untreated WS (58.9 vs. 31.2 g/kg DM; *p* < 0.001). OM content was not significantly affected (*p* = 0.256).

### 3.2. Structural Carbohydrate Composition of Cellulolytic Bacteria-Treated Ammoniated Wheat Straw

**Figure 1:**
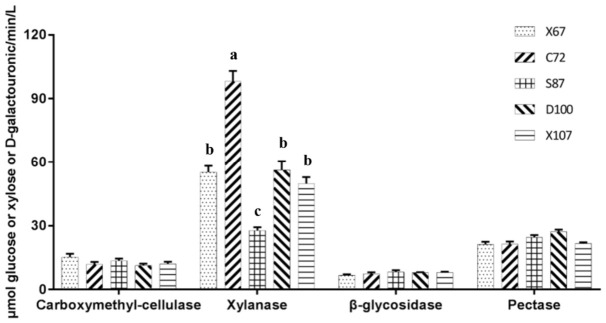
Enzyme activities of alkali-resistant cellulolytic bacteria. The values with different lowercase letters show significant difference among different treatments with the same storage time (*p* < 0.05).

Significant treatment × storage duration interactions (*p* < 0.05) were detected for DM, NDF, hemicellulose, and cellulose contents (Table 3). The bacterial treatments did not significantly affect DM content between 7 and 14 days; however, DM losses became more pronounced by 21 days. The X67, C72, and S87 treatments significantly (*p* < 0.05) reduced NDF, ADF, and cellulose contents after 7 and 14 days compared with the ammoniated control. The X107 treatment significantly reduced hemicellulose content by 8.7% compared with the ammoniated control at 7 days, while cellulose content remained relatively unchanged. The ADL content increased significantly over time for most treatments (*p* < 0.05), with S87 showing a 15.4% increase at 21 days. The CMCase, xylanase, and β-glycosidase activities of the five strains are shown in Figure 1, with C72 exhibiting the highest xylanase activity.

### 3.3. Gas Production Kinetics, Nutrient Degradation, and Fermentation Characteristics

The gas production rate did not show a significant treatment × storage duration interaction (Table 4); however, the main effect of treatment was significant (*p* < 0.05). All bacterial treatments, except X107, considerably increased potential gas production and 48-h methane production. The X107 treatment resulted in a 26.1% increase in potential gas production at 21 days. Both IVDMD and IVNDFD were significantly improved by bacterial inoculation, particularly at 7 days. The S87 treatment achieved the highest IVDMD (409 g/kg DM) and IVNDFD (628 g/kg DM) at 7 days, representing improvements of 14.9% and 32.5%, respectively, over the ammoniated control.

Although no significant treatment × storage duration interaction was detected for total VFA (Table 5), the main effect of treatment was significant (*p* < 0.05). All bacterial treatments significantly increased total VFA, acetate, and isobutyrate concentrations compared with the ammoniated control.

### 3.4. Ruminal Enzyme Activity and Microbial Analysis

The activities of CMCase, xylanase, and β-glycosidase are presented in Table 6. No significant treatment × storage duration interaction was observed for any of the enzyme activities; however, the main effect of treatment was significant (*p* < 0.05) for xylanase and β-glycosidase activities. Xylanase activity was consistently higher than CMCase and β-glycosidase activities across all treatments.

**Figure 2:**
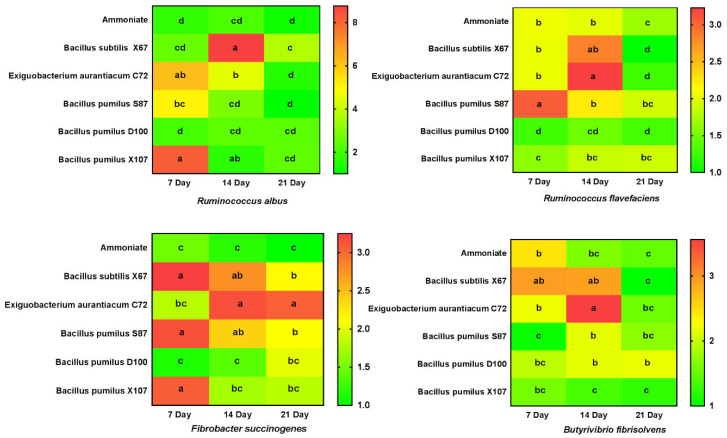
The relative proportion of ruminal cellulolytic bacteria after 48 h in vitro incubation of ammoniated wheat straw. The values with different lowercase letters show significant difference among different treatments with the same storage time (*p* < 0.05).

The relative proportions of ruminal cellulolytic bacteria after 48 h of in vitro incubation are shown in Figure 2. *F. succinogenes* was the most abundant cellulolytic species detected. Its relative abundance was significantly increased (*p* < 0.05) by the bacterial treatments compared with the ammoniated control. The abundance of total bacteria was not significantly affected by the D100 treatment; however, all other bacterial treatments increased (*p* < 0.05) the total bacterial population at 7 and 14 days compared with 21 days. The X107 treatment at 7 days and the X67 treatment at 14 days yielded the highest proportions of *R. albus* among the five bacterial inoculants tested.

## 4. Discussion

### 4.1. Role and Limitations of Ammoniation Pretreatment

The reduction in structural carbohydrates after ammoniation was consistent with previous studies [8,11], demonstrating the effectiveness of this treatment in disrupting the lignocellulosic matrix. The marked increase in crude protein content, attributed to the addition of non-protein nitrogen, is a well-recognised benefit of ammonia treatment [28]. However, the limited effect on acid detergent lignin content indicates that further research is needed to unlock the full potential of the cell wall. Although ammoniation can cleave some ester linkages and reduce cellulose crystallinity, it cannot effectively remove lignin. The presence of lignin physically hinders the attachment and degradation of cellulose and hemicellulose by rumen microorganisms [29,30]. Therefore, introducing cellulolytic bacteria that secrete fibrolytic enzymes (cellulases and hemicellulases) after ammoniation is a rational strategy to further disrupt the cell wall structure. It is important to clarify that the bacterial strains used in this study do not directly degrade lignin via hydrolytic enzymes; rather, their activity on cellulose and hemicellulose increases the porosity of the cell wall, facilitating subsequent ruminal microbial attachment and digestion. The observed increase in ADL content over time (Table 3) is a relative enrichment phenomenon resulting from the preferential consumption of more digestible carbohydrate fractions, rather than an indication of lignin synthesis or degradation.

### 4.2. Degradation Characteristics and Enzyme Activity

The distinct degradation patterns of the five bacterial strains highlight the importance of selecting strains according to the desired outcomes. The ability of X67, C72 and S87 to reduce the contents of neutral detergent fibre, acid detergent fibre and cellulose indicates a broad fibrolytic capacity. In contrast, the preferential degradation of hemicellulose by X107 (a 21% reduction) suggests a specific and potent xylanase activity. This substrate specificity is valuable because efficient hemicellulose removal can increase the porosity of the cell wall, thereby facilitating subsequent cellulose digestion by rumen microbes [31]. The high xylanase activity of strain C72 (Figure 1) is consistent with its good performance in hemicellulose reduction.

Notably, Figure 1 shows that the xylanase activity of C72 was significantly higher than that of the other strains; however, C72 did not achieve a greater hemicellulose reduction than X107 (Table 3: X107 decreased hemicellulose by 21%, whereas C72 decreased it by only approximately 6%). This apparent contradiction may be explained by the following factors. The in vitro enzyme activity assay uses a soluble substrate (xylan) and reflects the maximum catalytic rate of the enzyme under ideal conditions. In the actual straw matrix, enzymatic efficiency is also influenced by substrate accessibility, enzyme adsorption onto the solid interface, and synergistic interactions among different enzymes. X107 may possess a better ability to bind to lignocellulose or a more complete hemicellulolytic system (including auxiliary enzymes such as β-xylosidase and arabinofuranosidase), leading to superior degradation performance in the complex substrate. This finding suggests that a single enzyme activity indicator cannot fully predict the degradation performance of a strain on a complex substrate; a comprehensive evaluation should also include data on the actual degradation of structural carbohydrates.

The increase in acid detergent lignin content over time does not indicate lignin synthesis but is a well-documented phenomenon of relative enrichment, because the more digestible carbohydrate fractions are consumed by the inoculated bacteria [32]. This observation is consistent with the increased dry matter loss at 21 days (Table 3). Notably, the acid detergent lignin content of X67 and S87 at 21 days was 33% and 27% higher, respectively, than that at 7 days, whereas the lignin content of X107 showed little change throughout the storage period. This difference may be because X107 primarily attacks hemicellulose and has a relatively limited effect on cellulose, thereby slowing the consumption of digestible components and attenuating the relative enrichment of lignin.

### 4.3. Ruminal Fermentation Parameters and Methane Production

The improvements in in vitro gas production, methane production, IVDMD and IVNDFD are direct consequences of the increased substrate availability resulting from bacterial pretreatment. The significant treatment × storage duration interaction for digestibility parameters indicates that an optimal time window exists to maximise benefits. Beyond this window, the inoculated bacteria consume the released nutrients for their own maintenance, resulting in dry matter loss at 21 days [8]. The superior performance of the S87 treatment, especially the 32.5% increase in in vitro neutral detergent fibre digestibility at day 7, identifies this strain as the most promising candidate for improving fibre digestion.

It should be noted that the absolute concentrations of total VFA observed in this study (15.7–22.2 mmol/L) were lower than those typically reported in in vitro fermentation studies using mixed diets or high-quality forages (30–70 mmol/L). This is primarily attributable to the substrate characteristics: wheat straw is a low-quality, lignocellulose-rich forage with limited fermentable carbohydrates and crude protein content (31.2–58.9 g/kg DM). The in vitro system contained no concentrate supplementation or additional nitrogen sources beyond that present in the straw itself. Even with ammoniation, the nitrogen supply remained suboptimal for maximal microbial protein synthesis, which is a key determinant of VFA production [33]. In addition, the VFA concentrations reported were measured at the end of the 48 h incubation period, at which point a substantial proportion of the VFA produced may have been incorporated into microbial biomass or utilized for maintenance [34]. Despite these relatively low absolute values, the relative differences among treatments were consistent with the improvements observed in gas production and nutrient digestibility. Comparable VFA levels (15–25 mmol/L) from straw-based substrates in 48 h in vitro systems have been reported in previous studies [35], confirming that our data are within the expected range for this experimental setup. Importantly, all bacterial treatments, especially S87 and X67, significantly increased total VFA production compared with the ammoniated control, indicating that the inoculation effectively enhanced substrate fermentability.

Regarding the increase in methane production (Table 4), although it may be an environmental concern, it is an expected stoichiometric consequence of increased acetate and butyrate production in the rumen [36]. Acetate and butyrate are the main sources of hydrogen, and ruminal hydrogen is used by methanogens to produce methane. In the present study, the S87 and C72 treatments not only increased total volatile fatty acids but also significantly increased the proportions of acetate and butyrate (Table 5), which is consistent with the observed trend of increased methane production. It should be noted that the in vitro fermentation system is a closed system; methane produced in vitro cannot be eructated as it would be in the animal body. Therefore, in vitro methane production may overestimate the actual methane emissions in animal production. In addition, the higher methane production in this experiment may also be related to the increased availability of fermentable substrates: bacterial pretreatment released more fermentable carbohydrates, providing a more abundant methanogenic substrate for rumen microorganisms. Nevertheless, while aiming for higher feed digestibility, alleviating methane emissions through nutritional strategies (e.g., adding methane inhibitors or adjusting diet composition) remains a topic for further research.

### 4.4. Response Mechanisms of the Ruminal Microbial Community

The increased relative abundance of *F. succinogenes*, a primary cellulolytic bacterium, is a key finding because this species is known for its exceptional ability to digest crystalline cellulose [37,38]. The partial disruption of the cell wall by the exogenous bacterial additives likely supported the proliferation of *F. succinogenes* by making the cellulose fibrils more accessible. *F. succinogenes* is an obligate anaerobe that attaches tightly to the cellulose surface through a cellulosome-like structure on its cell membrane and efficiently degrades crystalline cellulose [38]. In the present study, the X67 and S87 treatments significantly increased the relative abundance of *F. succinogenes* (Figure 2), which is consistent with their excellent performance in improving neutral detergent fibre digestibility. The increase in total bacterial population and the strain-specific effects on *R. albus* further demonstrate a positive shift of the ruminal ecosystem towards a more efficient fibrolytic community. Notably, *R. albus* and *R. flavefaciens* are also important cellulolytic bacteria, but their ability to utilise hemicellulose is relatively weak [37]. In this study, the X107 treatment significantly increased the proportion of *R. albus* at day 7, which may be related to the preferential degradation of hemicellulose by X107, thereby exposing more binding sites for cellulose. The lack of effect of the D100 treatment on total bacterial abundance suggests that its mode of action may differ or that it is less potent under the tested conditions.

Beyond the laboratory-scale findings, several practical factors should be considered for the successful translation of this biological pretreatment strategy to farm-level application. First, the production cost of bacterial inoculants is a critical determinant of economic feasibility. The strains used in this study (*Bacillus* spp. and *Exiguobacterium* spp.) are generally amenable to low-cost cultivation using agricultural by-products as growth media, which could reduce production expenses [39]. Second, the stability and shelf-life of the inoculant under ambient storage conditions must be ensured to guarantee consistent performance in practice. Lyophilized or spore-based formulations, particularly for *Bacillus* strains, offer advantages in terms of long-term viability and ease of transportation [40]. Third, the scalability of the pretreatment process requires consideration of mixing uniformity, oxygen exposure, and temperature control during the 7- to 14-day storage period. In temperate regions, ambient temperatures (approximately 20 °C) may be suitable for spring and autumn application, whereas temperature fluctuations in summer or winter may necessitate insulated storage facilities [41]. Finally, the adoption of this technology by farmers will depend on the trade-off between improved feed digestibility and the additional labor and infrastructure requirements. Future research should focus on on-farm pilot trials to evaluate the techno-economic viability of this approach under real-world production conditions.

## 5. Conclusions

This study demonstrates that alkali-resistant cellulolytic bacterial inoculation further improves the feeding value of ammoniated wheat straw, with efficacy jointly regulated by strain type and storage duration. Inoculation for 7–14 days significantly enhanced in vitro nutrient digestibility and promoted the proliferation of ruminal cellulolytic microbiota, albeit with elevated methane production. Extending storage to 21 days led to excessive nutrient consumption by inoculant strains, compromising substrate preservation. Among all tested strains, *Bacillus pumilus* S87 delivered the best performance when applied for 7 days: it outperformed the ammoniation-only control by 14.9% in IVDMD and 32.5% in IVNDFD, representing the most promising treatment for practical application. Notably, these findings were derived from in vitro ruminal fermentation assays; follow-up in vivo trials are warranted to validate on-farm performance, alongside the development of complementary methane mitigation strategies to balance feed efficiency gains with environmental sustainability.

## Figures and Tables

**Table 1:** PCR primers for real-time PCR assay.

Target Species	Forward/Reverse	Sequence of Primer (5′ → 3′)
Total bacteria	F	CGGTGAATACGTTCYCGG
	R	GGWTACCTTGTTACGACTT
*Ruminococcus albus*	F	CCCTAAAAGCAGTCTTAGTTCG
	R	CCTCCTTGCGGTTAGAACA
*Ruminococcus flavefaciens*	F	ACCGCATAAGCGCACGGA
	R	CGGGTCCATCTTGTACCGATAAAT
*Fibrobacter succinogenes*	F	GGCGGGATTGAATGTACCTTGAGA
	R	TCCGCCTGCCCCTGAACTATC
*Butyrivibrio fibrisolvens*	F	ACCGCATAAGCGCACGGA
	R	CGGGTCCATCTTGTACCGATAAAT

**Table 2:** Effect of ammoniation on chemical composition of wheat straw.

Item ^1^	Treatment		SEM	*p*-Value
	Control	Ammoniate		
DM (g/kg FW)	950	659	20.1	<0.001
OM (g/kg DM)	925	922	4.21	0.256
CP (g/kg DM)	31.2	58.9	0.44	<0.001
NDF (g/kg DM)	749	710	8.74	<0.001
ADF (g/kg DM)	525	496	8.37	<0.001
ADL (g/kg DM)	141	139	4.95	0.053
Hemicellulose (g/kg DM)	224	214	0.61	0.045
Cellulose (g/kg DM)	345	317	4.03	0.015

^1^ ADF, acid detergent fiber; ADL, acid detergent lignin; DM, dry matter; FW, fresh weight; NDF, neutral detergent fiber; OM, organic matter, CP, Crude protein.

**Table 3:** Effect of alkali-resistant cellulolytic bacteria on structural carbohydrate composition of ammoniated wheat straw.

Items	Storage Days	Treatment ^1^						SEM	*p*-Value ^2^		
		A	X67	C72	S87	D100	X107		T	D	T × D
DM (g/kg FW)	7 d	602 ^a^	602 ^a^	595 ^b^	600 ^a^	597 ^ab^	601 ^a^				
	14 d	598 ^ab^	597 ^ab^	592 ^b^	593 ^b^	590 ^b^	598 ^ab^	1.36	0.013	0.001	0.026
	21 d	579 ^c^	582 ^c^	578 ^c^	578 ^c^	572 ^d^	578 ^c^				
OM (g/kg DM)	7 d	916 ^c^	915 ^c^	921 ^b^	919 ^b^	925 ^a^	921 ^b^				
	14 d	916 ^c^	914 ^c^	919 ^b^	920 ^b^	926 ^a^	919 ^b^	0.51	0.001	0.091	0.051
	21 d	914 ^c^	915 ^c^	920 ^b^	917 ^bc^	920 ^b^	916 ^c^				
NDF (g/kg DM)	7 d	740 ^b^	715 ^c^	712 ^c^	713 ^c^	725 ^bc^	712 ^c^				
	14 d	738 ^b^	721 ^c^	723 ^c^	721 ^c^	726 ^bc^	717 ^c^	2.22	<0.001	<0.001	0.004
	21 d	745 ^ab^	731 ^b^	735 ^b^	756 ^a^	745 ^ab^	760 ^a^				
ADF (g/kg DM)	7 d	510 ^b^	474 ^ef^	480 ^e^	470 ^f^	486 ^de^	502 ^c^				
	14 d	512 ^b^	474 ^ef^	485 ^de^	476 ^ef^	490 ^d^	505 ^c^	2.36	0.035	<0.001	0.133
	21 d	508 ^bc^	508 ^bc^	516 ^ab^	505 ^c^	520 ^a^	511 ^b^				
ADL (g/kg DM)	7 d	115 ^e^	121 ^de^	137 ^bc^	130 ^c^	133 ^c^	127 ^cd^				
	14 d	143 ^b^	144 ^b^	144 ^b^	146 ^b^	143 ^b^	126 ^cd^	2.03	0.033	<0.001	0.075
	21 d	143 ^b^	161 ^a^	146 ^b^	165 ^a^	129 ^cd^	141 ^bc^				
Hemicellulose (g/kg DM)	7 d	230 ^b^	241 ^ab^	232 ^b^	243 ^ab^	240 ^ab^	210 ^c^				
	14 d	226 ^b^	227 ^b^	224 ^bc^	209 ^c^	225 ^bc^	205 ^c^	2.10	0.081	0.002	0.009
	21 d	237 ^ab^	223 ^bc^	228 ^b^	251 ^a^	225 ^bc^	249 ^a^				
Cellulose (g/kg DM)	7 d	352 ^a^	313 ^c^	303 ^cd^	300 ^d^	313 ^c^	335 ^ab^				
	14 d	336 ^ab^	312 ^c^	315 ^c^	323 ^bc^	328 ^b^	341 ^ab^	2.71	<0.001	<0.001	0.002
	21 d	335 ^ab^	317 ^c^	331 ^b^	322 ^bc^	330 ^b^	342 ^ab^				

^1^ A: ammoniate; X67: *Bacillus subtilis* isolated from the faeces of rhinoceros; C72: *Exiguobacterium aurantiacum* isolated from the faeces of giraffe; S87: *Bacillus pumilus* isolated from decaying wood; D100: *Bacillus pumilus* isolated from the faeces of elephant; X107: *Bacillus pumilus* isolated from the faeces of rhinoceros. The same as below. ^2^ T: treatment; D: storage days; T × D: interaction between treatment and storage days. The values with different lowercase letters show significant difference among different treatments with the same storage time (*p* < 0.05).

**Table 4:** Effect of alkali-resistant cellulolytic bacteria on gas production kinetics, in vitro dry matter digestibility (IVDMD) and neutral detergent fibre (IVNDFD) of ammoniated wheat straw.

Items	Storage Days	Treatment ^1^						SEM	*p*-Value ^2^		
		A	X67	C72	S87	D100	X107		T	D	T × D
Potential gas production (mL/g)	7 d	63.8 ^d^	120 ^a^	127 ^a^	131 ^a^	85.0 ^bc^	63.5 ^d^				
	14 d	57.0 ^d^	62.7 ^d^	79.9 ^c^	74.6 ^c^	98.9 ^b^	83.1 ^bc^	3.01	<0.001	<0.001	<0.001
	21 d	57.9 ^d^	99.7 ^b^	93.5 ^b^	93.9 ^b^	72.6 ^c^	57.9 ^d^				
Rate of gas production (10^−3^/h)	7 d	0.048 ^e^	0.053 ^d^	0.058 ^cd^	0.062 ^c^	0.049 ^e^	0.068 ^b^				
	14 d	0.062 ^c^	0.067 ^b^	0.054 ^d^	0.052 ^de^	0.046 ^ef^	0.041 ^f^	0.002	0.001	0.740	0.058
	21 d	0.059 ^cd^	0.054 ^d^	0.074 ^a^	0.072 ^a^	0.074 ^a^	0.074 ^a^				
48 h methane production (mL/g)	7 d	12.8 ^d^	22.3 ^c^	36.7 ^a^	31.4 ^b^	32.6 ^ab^	13.2 ^d^				
	14 d	13.3 ^d^	27.3 ^bc^	27.4 ^bc^	22.4 ^c^	25.3 ^bc^	20.1 ^c^	1.12	0.004	<0.001	<0.001
	21 d	13.7 ^d^	34.4 ^a^	20.7 ^c^	26.3 ^bc^	26.5 ^bc^	15.0 ^d^				
IVDMD (g/kg DM)	7 d	356 ^d^	397 ^ab^	402 ^a^	409 ^a^	372 ^bc^	365 ^c^				
	14 d	332 ^e^	316 ^f^	328 ^e^	336 ^e^	360 ^cd^	367 ^c^	4.15	<0.001	0.011	0.001
	21 d	338 ^e^	384 ^b^	374 ^bc^	384 ^b^	355 ^d^	339 ^e^				
IVNDFD (g/kg DM)	7 d	474 ^d^	536 ^bc^	565 ^b^	628 ^a^	517 ^c^	423 ^f^				
	14 d	418 ^fg^	389 ^g^	457 ^e^	470 ^de^	481 ^d^	463 ^e^	8.01	<0.001	<0.001	<0.001
	21 d	440 ^f^	508 ^c^	440 ^f^	498 ^c^	405 ^fg^	434 ^f^				

^1^ A: ammoniate; X67: *Bacillus subtilis* isolated from the faeces of rhinoceros; C72: *Exiguobacterium aurantiacum* isolated from the faeces of giraffe; S87: *Bacillus pumilus* isolated from decaying wood; D100: *Bacillus pumilus* isolated from the faeces of elephant; X107: *Bacillus pumilus* isolated from the faeces of rhinoceros. The same as below. ^2^ T: treatment; D: storage days; T × D: interaction between treatment and storage days. The values with different lowercase letters show significant difference among different treatments with the same storage time (*p* < 0.05).

**Table 5:** Effect of alkali-resistant cellulolytic bacteria on ruminal VFA production (mmol/L) after 48 h in vitro incubation of ammoniated wheat straw.

Items	Storage Days	Treatment ^1^						SEM	*p*-Value ^2^		
		A	X67	C72	S87	D100	X107		T	D	T × D
Total VFA	7 d	17.8 ^cd^	19.7 ^b^	20.6 ^ab^	21.6 ^a^	16.4 ^d^	16.5 ^d^				
	14 d	16.0 ^d^	17.4 ^d^	17.9 ^cd^	19.3 ^b^	20.2 ^ab^	19.0 ^bc^	0.304	0.042	0.087	0.156
	21 d	15.7 ^d^	22.2 ^a^	18.7 ^c^	19.8 ^b^	19.1 ^bc^	18.6 ^c^				
Acetate	7 d	12.8 ^c^	13.5 ^bc^	13.7 ^b^	14.5 ^b^	10.9 ^d^	11.4 ^cd^				
	14 d	11.4 ^cd^	11.9 ^cd^	12.7 ^c^	13.6 ^bc^	13.8 ^bc^	13.5 ^bc^	0.219	0.041	0.052	0.112
	21 d	11.1 ^d^	16.5 ^a^	13.6 ^bc^	14.9 ^ab^	14.0 ^b^	13.7 ^bc^				
Propionate	7 d	3.26 ^cd^	3.62 ^bc^	3.96 ^ab^	4.12 ^a^	3.04 ^e^	3.30 ^cd^				
	14 d	3.05 ^e^	3.14 ^de^	3.20 ^d^	3.47 ^c^	3.71 ^b^	3.20 ^d^	0.065	0.043	0.117	0.092
	21 d	2.97 ^e^	3.71 ^b^	3.16 ^de^	3.16 ^de^	3.18 ^de^	3.05 ^e^				
Isobutyrate	7 d	0.144 ^e^	0.258 ^b^	0.312 ^a^	0.310 ^a^	0.301 ^a^	0.190				
	14 d	0.146 ^e^	0.263 ^ab^	0.208 ^c^	0.234 ^bc^	0.275 ^ab^	0.248 ^b^	0.011	0.009	0.012	0.550
	21 d	0.149 ^e^	0.192 ^cd^	0.187 ^d^	0.176 ^de^	0.210 ^c^	0.175 ^de^				
Butyrate	7 d	1.21 ^de^	1.63 ^b^	1.86 ^a^	1.88 ^a^	1.51 ^bc^	1.19 ^de^				
	14 d	1.04 ^e^	1.43 ^c^	1.29 ^d^	1.41 ^c^	1.71 ^ab^	1.49 ^bc^	0.047	0.008	0.046	0.279
	21 d	1.05 ^e^	1.38 ^cd^	1.29 ^d^	1.22 ^de^	1.21 ^de^	1.20 ^de^				
Isovalerate	7 d	0.238 ^ef^	0.431 ^bc^	0.526 ^a^	0.478 ^ab^	0.438 ^b^	0.289 ^d^				
	14 d	0.234 ^ef^	0.429 ^bc^	0.335 ^cd^	0.382 ^c^	0.451 ^b^	0.415 ^bc^	0.020	0.023	0.163	0.559
	21 d	0.248 ^ef^	0.217 ^f^	0.298 ^d^	0.269 ^de^	0.332 ^cd^	0.279 ^de^				
Valerate	7 d	0.132 ^f^	0.194 ^cd^	0.251 ^a^	0.231 ^ab^	0.201 ^c^	0.149 ^de^				
	14 d	0.129 ^f^	0.191 ^cd^	0.158 ^de^	0.177 ^d^	0.220 ^bc^	0.201 ^c^	0.007	0.039	0.177	0.373
	21 d	0.143 ^ef^	0.160 ^de^	0.153 ^de^	0.141 ^ef^	0.154 ^de^	0.147 ^e^				

^1^ A: ammoniate; X67: *Bacillus subtilis* isolated from the faeces of rhinoceros; C72: *Exiguobacterium aurantiacum* isolated from the faeces of giraffe; S87: *Bacillus pumilus* isolated from decaying wood; D100: *Bacillus pumilus* isolated from the faeces of elephant; X107: *Bacillus pumilus* isolated from the faeces of rhinoceros. The same as below. ^2^ T: treatment; D: storage days; T × D: interaction between treatment and storage days. The values with different lowercase letters show significant difference among different treatments with the same storage time (*p* < 0.05).

**Table 6:** Effect of alkali-resistant cellulolytic bacteria on ruminal cellulase activity (IU/L) after 48 h in vitro incubation of ammoniated wheat straw.

Items	Storage Days	Treatment ^1^						SEM	*p*-Value ^2^		
		A	X67	C72	S87	D100	X107		T	D	T × D
Carboxymethyl-cellulase	7 d	8.56	9.51	8.51	8.36	8.54	8.83				
	14 d	8.89	8.68	8.61	8.79	8.59	8.87	0.062	0.074	0.506	0.300
	21 d	8.95	8.75	8.97	8.90	8.84	8.52				
Xylanase	7 d	37.3 ^b^	40.2 ^a^	38.5 ^ab^	41.8 ^a^	36.6 ^bc^	39.1 ^ab^				
	14 d	32.5 ^cd^	35.6 ^c^	37.0 ^b^	41.7 ^a^	36.9 ^b^	34.1 ^c^	0.801	0.034	0.256	0.231
	21 d	33.3 ^cd^	39.3 ^ab^	40.1 ^a^	38.4 ^ab^	30.5 ^d^	37.2 ^b^				
β-glycosidase	7 d	12.4 ^ab^	13.0 ^a^	12.4 ^ab^	12.9 ^a^	12.1 ^b^	12.0 ^b^				
	14 d	11.6 ^bc^	11.4 ^c^	11.6 ^bc^	11.7 ^bc^	11.3 ^c^	11.7 ^bc^	0.129	0.005	0.103	0.424
	21 d	11.8 ^b^	12.4 ^ab^	12.4 ^ab^	11.7 ^bc^	10.0 ^d^	11.8 ^b^				

^1^ A: ammoniate; X67: *Bacillus subtilis* isolated from the faeces of rhinoceros; C72: *Exiguobacterium aurantiacum* isolated from the faeces of giraffe; S87: *Bacillus pumilus* isolated from decaying wood; D100: *Bacillus pumilus* isolated from the faeces of elephant; X107: *Bacillus pumilus* isolated from the faeces of rhinoceros. The same as below. ^2^ T: treatment; D: storage days; T × D: interaction between treatment and storage days. The values with different lowercase letters show significant difference among different treatments with the same storage time (*p* < 0.05).

## Data Availability

The original contributions presented in the study are included in the article.
